# Silver Nanoparticles Functionalized Nanosilica Grown over Graphene Oxide for Enhancing Antibacterial Effect

**DOI:** 10.3390/nano12193341

**Published:** 2022-09-25

**Authors:** Qui Quach, Tarek M. Abdel-Fattah

**Affiliations:** Applied Research Center at Thomas Jefferson National Accelerator Facility, Department of Molecular Biology and Chemistry, Christopher Newport University, Newport News, VA 23606, USA

**Keywords:** silver nanoparticles, nanosilica, graphene oxide composites, antibacterial effect, *Escherichia coli*, bacillus subtilis

## Abstract

The continuous growth of multidrug-resistant bacteria due to the overuse of antibiotics and antibacterial agents poses a threat to human health. Silver nanoparticles, silica-based materials, and graphene-based materials have become potential antibacterial candidates. In this study, we developed an effective method of enhancing the antibacterial property of graphene oxide (GO) by growing nanosilica (NS) of approximately 50 nm on the graphene oxide (GO) surface. The structures and compositions of the materials were characterized through powdered X-ray diffraction (P-XRD), transmission electron microscopy (TEM), scanning electron microscopy coupled with energy dispersive X-ray spectroscopy (SEM-EDS), ultraviolet–visible spectroscopy (UV–VIS), dynamic light scattering (DLS), Raman spectroscopy (RM), Fourier-transform infrared spectroscopy (FTIR), Brunauer–Emmet–Teller (BET) surface area, and pore size determination. The silver nanoparticles (AgNPs) with an average diameter of 26 nm were functionalized on the nanosilica (NS) surface. The composite contained approximately 3% of silver nanoparticles. The silver nanoparticles on nanosilica supported over graphene oxide (GO/NS/AgNPs) exhibited a 7-log reduction of *Escherichia coli* and a 5.2-log reduction of *Bacillus subtilis* within one hour of exposure. Both GO/NS and GO/NS/AgNPs exhibited substantial antimicrobial effects against *E. coli* and *B. subtilis*

## 1. Introduction

Multidrug-resistant bacteria have become a global health threat in the 21st century [1]. Antibiotic resistance has caused common infections to become more difficult to treat and has led to expensive healthcare costs [1]. In order to reduce the dependency on antibiotic treatments which are ineffective against resistant bacterial strains, various studies have been conducted to develop novel materials to treat and prevent the growth of these pathogens [2,3,4,5]. Among those materials, silver nanoparticles appear to have the most potential and be the most attractive solution due to their antibacterial properties [6,7]. However, some studies have shown that bacteria can develop resistance to silver nanoparticles after repeated exposure [8,9]. The resistant strains produced adhesive flagellum that caused the nanoparticles to aggregate, resulting in a reduction in the surface area and antibacterial effect [8,9].

In previously reported studies, support templates or capping agents were often used to improve the size, shape, and stability of nanoparticles [10,11,12,13,14]. It is important to control the surface properties, morphology, and functionality for bio-medical applications [15,16,17]. Among the materials, silica-based and graphene materials are excellent support templates for enhancing antimicrobial activity [18,19]. These materials are well-known in medicine and biotechnology [19,20,21]. In a study from Liu et al., silver nanoparticles were loaded on to the graphene oxide and a large silicate template, but the synthesis process required heating which caused the graphene oxide to reduce [22]. Previous research reported that graphene oxide had a higher antibacterial ability than that of the reduced form [23]. Additionally, the large mesoporous silica template was less effective than small-sized silica nanoparticles in improving the antimicrobial property of the composite [24]. Based on different review studies, the combination of graphene oxide, nanosilica, and silver nanoparticles was neither synthesized nor tested for antibacterial activity [25,26]. Furthermore, the method which we applied in this study to grow nanosilica on graphene oxide was different from other reported methods [27,28,29]. In a Yang et al. study, the SiO_2_ was functionalized with an amine group and then loaded on to the graphene oxide; the pH of the synthesis process was required to be 7 for the coupling process to be successful [27]. Another study by Kou and Gao grew the silica nanoparticles on the graphene oxide with the assistance of ammonia, but the resulting product had to be stored in alcohol [28]. In our study, our product was formed in a basic condition and was stored without using any solvent.

In our study, we grew the nanosilica (NS) on graphene oxide (GO) with a modified method to avoid the formation of a large silicate sandwich-like layer. The silver nanoparticles (AgNPs) were then loaded on to the nanosilica (NS) to form the composite (GO/NS/AgNPs). The structure and composition of GO/NS and GO/NS/AgNPs composites were characterized via P-XRD, TEM, SEM-EDS, UV–VIS, DLS, Raman spectroscopy, FTIR, and BET surface area measurements. The antibacterial ability of GO/NS and GO/NS/AgNPs against *Escherichia coli* (*E. coli*) and *Bacillus subtilis* (*B. subtilis*) was examined using the spread plate method and zone inhibition test.

## 2. Experimental

### 2.1. Synthesis of Nanosilica Grown on Graphene Oxide

Graphene oxide (GO) was synthesized by following the method reported by Bhawal et al. [30]. We modified the Liu et al. method to synthesize nanosilica (NS) on GO [31]. A total of 50 mL of GO solution was prepared at a 2:1 ratio of GO and deionized water (DI, 18 MΩ). An amount of 500 mg of CTAB (Acros Organics, Geel, Belgium, 99%) and 20 mg of sodium hydroxide (Sigma Aldrich, St. Louis, MO, USA, 97%) were mixed together with the GO solution through sonication. Then, 0.5 mL of tetraethyl orthosilicate (TEOS) (Tokyo Chemical Industry, Tokyo, Japan, 96%) was added to the above mixture and heated in a shaking water bath overnight at 40 °C. The solution was refluxed in 50 mL of hydrogen chloride and ethanol (1% *v*/*v*) for 3 h to remove the CTAB template. The collected GO/NS was washed with ethanol and deionized water (DI, 18 MΩ).

### 2.2. Synthesis of Silver Nanoparticles and Silver Nanoparticles Supported over Nanosilica/Graphene Oxide Composites

A total of 50 mL of silver nitrate (Sigma-Aldrich, Burlington, Massachusetts, 99%) (0.001 M) was prepared and heated until boiling. Then, 5 mL of 1% w/w sodium citrate was added slowly to the boiling solution. After the solution turned a yellow color, the solution was cooled to room temperature (293 K) and then centrifuged at 10,000 rpm for 10 min to remove unreacted solutes. Then, 2 mL of colloidal silver nanoparticles (AgNPs) was added to 10 mg of GO/NS. The mixture was sonicated for 15 min and then dried at ambient temperature (293 K) to obtain silver nanoparticles supported over GO/NS (GO/NS/AgNPs).

### 2.3. Characterization

The silver nanoparticles were examined via ultraviolet–visible spectroscopy (UV–VIS, Shimadzu UV-2600). Approximately 3.5 mL of colloidal AgNPs was added to a cuvette. The sample was then scanned from 600 nm to 300 nm.

The composite’s crystal structures were characterized by using powdered X-ray diffraction (P-XRD, Rigaku Miniflex II, Cu Kα X-ray, nickel filters). Each sample was spread flat on a sample holder. The P-XRD of each material was scanned from 5° to 90°.

The functional groups of GO, GO/NS (before and after removing CTAB), and GO/NS/AgNPs were confirmed through Fourier-transform infrared spectroscopy (FTIR, Shimadzu IR-Tracer 100). A small amount of the sample was placed on the ATR attachment of FTIR, and then each sample was scanned from 4000 cm^−1^ to 500 cm^−1^. The chemical structures of GO/NS and GO/NS/AgNPs were also verified by using a Raman microscope and spectrometer (Renishaw, ISC3-1233). The samples were spread flat on the sample slides. The Raman microscope was used to locate the samples as well as adjusting the resolution. After that, each sample was measured from 3000 cm^−1^ to 150 cm^−1^.

In TEM analysis, each sample was dispersed and sonicated in 5 mL of DI water. A few drops of the solution were added to a Cu grid (300 mesh). The grid was dried in the oven at 80 °C overnight. Then, each grid was scanned via transmission electron microscopy (TEM, JEM-2100F).

The size and dispersity of graphene, silicate nanoparticles, and silver nanoparticles were analyzed via dynamic light scattering (DLS, Brookhaven Instrument, Nanobrook 90Plus). Before analyzing via DLS, the sample was prepared by mixing it with DI water (1 mg/mL) and sonicating it for an hour to fully dissolve the sample. Then, the prepared sample was filtered to avoid undissolved particles. The solutions were then added to the quartz curvette and then scanned through DLS.

The surface structure image of GO/NS/AgNPs was obtained via scanning electron microscopy (SEM, JEOL JSM-6060LV). The weight ratios among elements and elemental mapping images were determined through energy-dispersive X-ray spectroscopy (EDS, Thermo Scientific UltraDry). The nitrogen adsorption–desorption isotherm, BET surface area, and pore size of each composite were measured by using an Accelerated Surface Area and Porosimetry System (Micromeritics -ASAP 2020) at 77.4 K. The composites were degassed for approximately 12 h before being analyzed with the ASAP system.

### 2.4. Antibacterial Activity Assay

The method by Rajapaksha et al. was applied to test the antibacterial ability of GO/NS and GO/NS/AgNPs against *E. coli* and *B. subtilis* [32]. All tools and glassware were sterilized by autoclaving at 120 °C. The sterile LB broth medium was inoculated with either E. coli or B. subtilis overnight at 37 °C in a shaking incubator. A total of 10 mg of the composite was mixed with 9 mL of 1x PBS buffer. The control only contained 9 mL of 1x PBS buffer. An amount of 1 mL of bacterial solution was transferred to both the control tube and the tubes that contained composites. A serial dilution method was conducted by transferring 1 mL of stock solution from the control tubes or composite tubes to tubes containing 9 mL of PBS solution until it reached the fifth diluent. Then, 0.1 mL of the solution in the diluent was transferred and spread on agar plates which were incubated overnight at 37 °C. The colony forming unit (CFU) was determined and calculated via CFU scope v1.6 software. The images of colony forming unit (CFU) plates for the control and each material are shown in the supplementary material Figures S1 and S2. The test was repeated twice. The data were statistically analyzed by ANOVA.

The zone inhibition test against *E. coli* and *B. subtilis* followed the Kirby–Bauer disk diffusion susceptibility test protocol. An average of 5.5 mg of each sample (GO/NS, GO/NS/AgNPs) was loaded on to the sterile disk. The control disk only received sterile DI water. The disks were placed on to the agar plates that were inoculated with bacteria and streaked. The plates were incubated overnight at 37 °C. The zone of inhibition of each composite disk was measured using a caliper and compared to the control disk. The images of the disk diffusion experiment for the control and each material are depicted in the Supplementary Material Figure S3.

## 3. Result and Discussion

**Figure 1:**
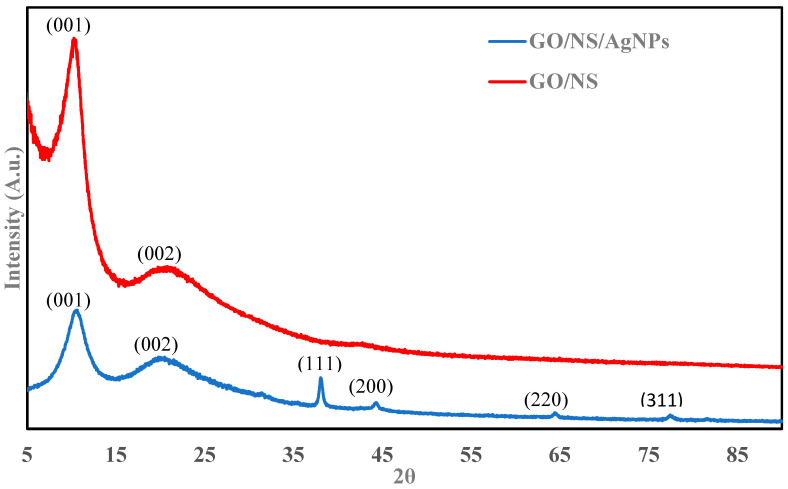
P-XRD of GO/NS/AgNPs and GO/NS.

**Figure 2:**
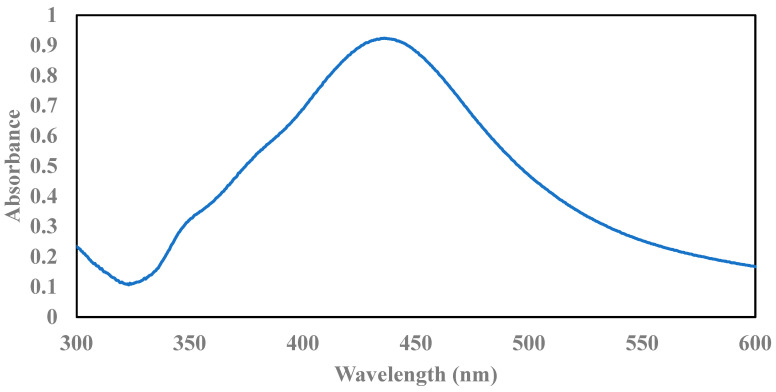
UV–VIS spectrum of AgNPs.

Figure 1 shows the P-XRD spectra of GO/MS and GO/NS/AgNPs. The peaks at 10° and 20° are indicative of the (001) plane of GO [33]. The nanosilica (NS) had peaks at 10° and 21° which corresponded with the (001) and (002) planes (JCPDS 29-0085). The planes were consistent with other literature [33,34]. After coupling with the AgNPs, the characteristic peaks of the AgNPs can be found at 38°, 44°, 64°, and 77°. These peaks corresponded with the (111), (200), (220), and (311) lattice planes of AgNPs (JCPDS 65-2871). The results were consistent with previously reported studies [35,36]. The UV–VIS spectrum in Figure 2 shows absorbance at 437 nm, which confirms the presence of AgNPs [35].

**Figure 3:**
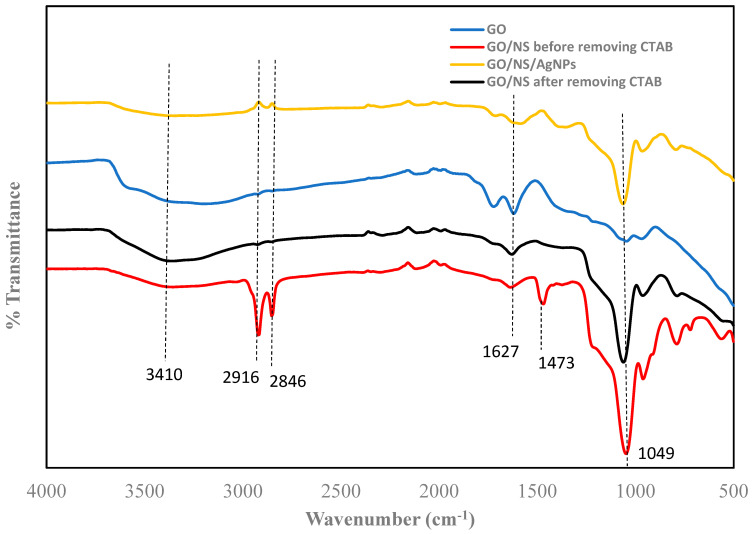
FTIR of GO, GO/NS before and after removing CTAB, and GO/NS/AgNPs.

Figure 3 depicts the functional groups of GO, GO/NS, and GO/NS/AgNPs. GO showed broad peaks at 3410 cm^−1^ which corresponds with the O-H stretching group. The peaks at 1627 cm^−1^ and 1049 cm^−1^ indicate the presence of C=C and C-O stretching groups. These results were consistent with the characterization of GO reported in previous literature [37,38]. The peak at 1049 cm^−1^ of GO/NS and GO/NS/AgNPs was sharper due to the presence of Si-O-Si. This phenomenon was reported in a Nodeh et al. study [39]. Before removing the CTAB template, the material showed bands at 2916 cm^−1^ and 2846 cm^−1^ which were contributed to by C-H stretching vibration of the methyl and methylene group of CTAB [40]. The small band at 1473 cm^−1^ was attributed to the C-H bending vibration of CTAB [40]. After removing the CTAB, those three bands (2916 cm^−1^, 2846 cm^−1^, and 1473 cm^−1^) disappeared. The result indicated that the reflux method completely eliminated the CTAB.

**Figure 4:**
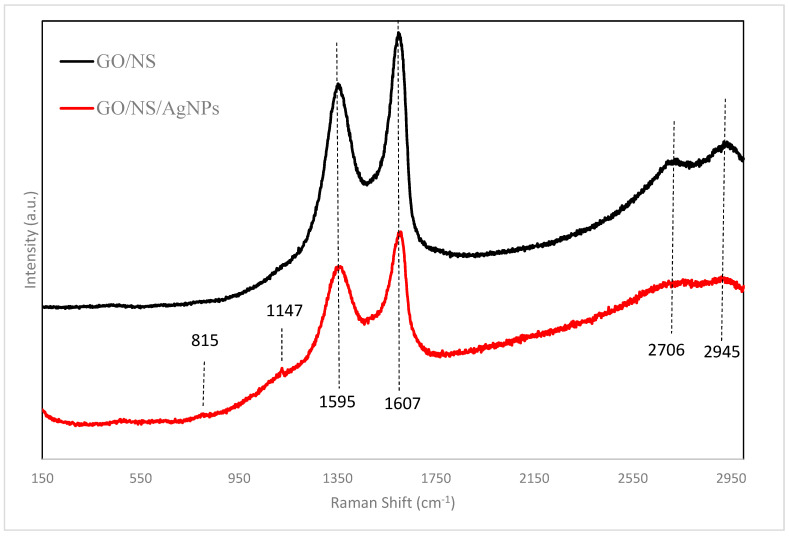
Raman spectra of GO/NS and GO/NS/AgNPs.

The chemical structures of GO/NS and GO/NS/AgNPs were further analyzed using Raman spectroscopy. Figure 4 shows the Raman spectra of GO/NS and GO/NS/AgNPs. The G band and the D band of GO were observed at 1607 cm^−1^ and 1360 cm^−1^. In a study of Perumbilavil et al., the G band of GO ranged from 1607 cm^−1^ to 1595 cm^−1^, while the D band of GO ranged from 1365 cm^−1^ to 1355 cm^−1^ [41]. The G band was contributed to by the C-C stretching. The D bands formed and sharpened after the graphite was oxidized and caused a reduction in the sp^2^ carbon domain [34]. Two bands at 2943 cm^−1^ and 2716 cm^−1^ were attributed to the silicate nanoparticles. In the research of Carboni et al., these mesoporous silicates exhibited bands at 2945 cm^−1^ and 2706 cm^−1^ [42]. The bands of silver nanoparticles were tiny due to their low concentration within the composite. The peaks at 1147 cm^−1^ and 815 cm^−1^ corresponded to the C-H bending of AgNPs’ capping agent. The Raman spectrum in a study by Kora et al. showed the C-H groups in their synthesized nanoparticles appeared in the range of 1165 cm^−1^–803 cm^−1^ [43].

**Figure 5:**
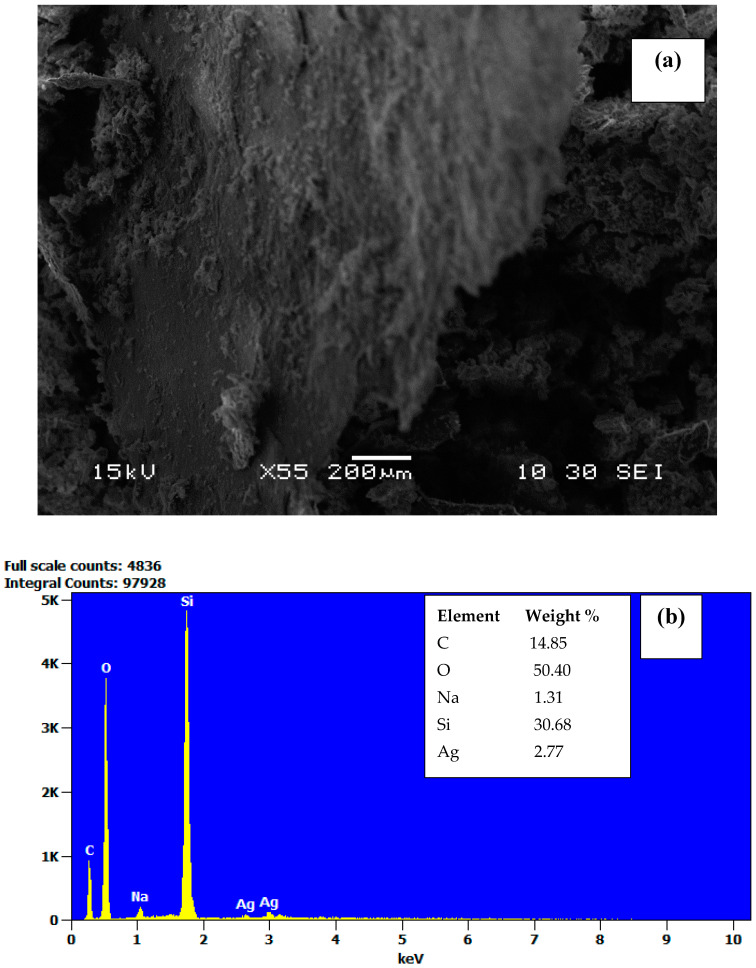
SEM image of (**a**) GO/NS/AgNPs (scale bar of 200 μm) and (**b**) EDS of GO/NS/AgNPs.

**Figure 6:**
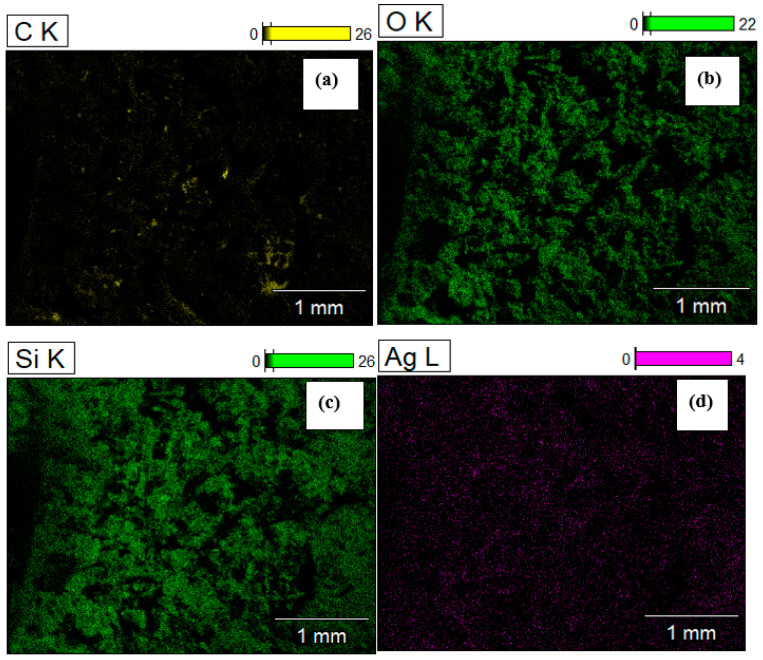
Elemental mapping of carbon (**a**), oxygen (**b**), silica (**c**), and silver (**d**) of GO/NS/AgNPs.

The SEM image (Figure 5a) depicts the micrograph of GO/NS/AgNPs. The result of EDS (Figure 5b) shows that the composite contained approximately 3% (wt %) of AgNPs. The ratios of AgNPs to GO and NS were 1:5 and 1:10, respectively. The elemental mapping images across the surface of GO/NS/AgNPs are represented in Figure 6, which shows that both Si (silica nanoparticles) (Figure 6c) and Ag (silver nanoparticles) (Figure 6d) were homogeneously distributed. Figure 6b,c depict similar distribution patterns which were due to the Si-O-Si bonding of the silicate nanoparticles.

**Figure 7:**
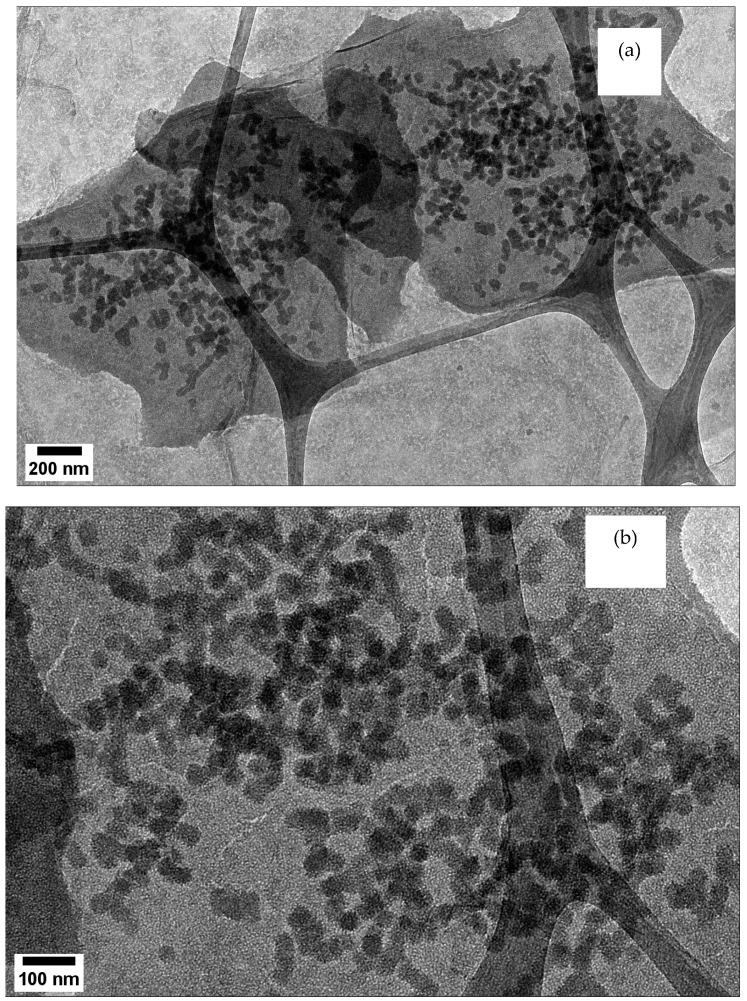

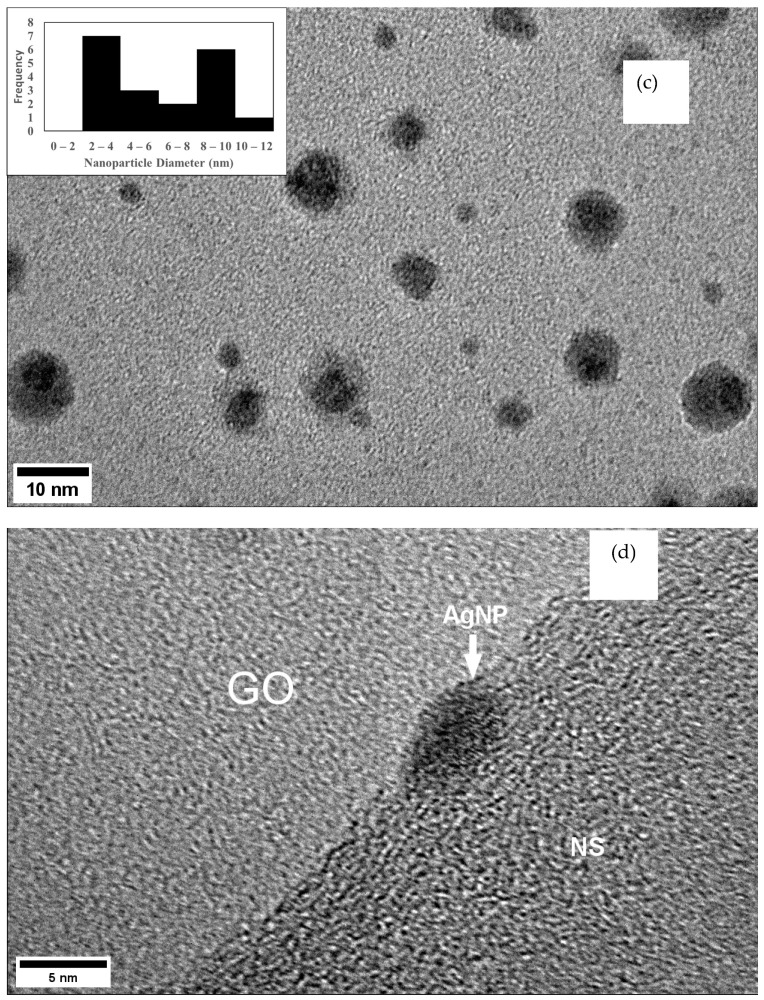
(**a**) TEM depicts NS grown on GO (scale bar of 200 nm); (**b**) TEM of NS grown on GO (scale bar of 100 nm); (**c**) TEM depicts the distribution of AgNPs on the nanosilica (scale bar of 10 nm); (**d**) TEM shows single AgNP on the nanosilica (scale bar of 5 nm).

**Figure 8:**
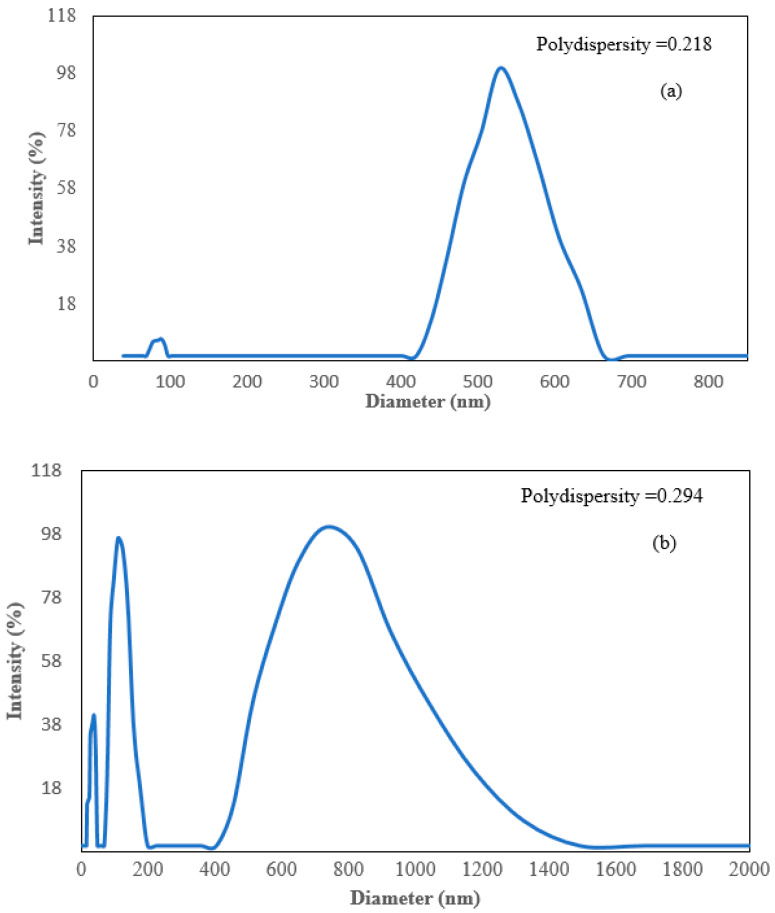
Dynamic light scattering (DLS) spectrums of (**a**) GO/NS and (**b**) GO/NS/AgNPs.

Figure 7a,b depict the TEM of silicate nanoparticles grown on graphene oxide. Figure 7c,d show the TEM images of AgNPs dispersed on the NS at scales of 20 nm and 10 nm, respectively. The images indicate that AgNPs were well-dispersed and exhibited no aggregation. It appeared that the materials highly supported and evenly distributed the AgNPs. The particle size distribution of GO/NS and GO/NS/AgNPs is represented in Figure 8a,b. Figure 8a consisted of two peaks which represented the silicate nanoparticles and graphene oxide. Silicate nanoparticles and graphene oxide had average sizes of 85 nm and 527 nm. In Figure 8b, there were three peaks that were contributed to by silver nanoparticles, silicate nanoparticles, and graphene oxide. The first peak indicated that silver nanoparticles had an average diameter of 26 nm. The second and third peaks represented the silicate nanoparticles and graphene oxide with sizes of 109 nm and 731 nm, respectively. Figure 8a,b indicated that the GO had a size range from 527 nm to 731 nm, while the size of silicate nanoparticles ranged from 85 nm to 109 nm. The polydispersity indexes of GO/NS and GO/NS/AgNPs were reported to be 0.218 and 0.294, respectively. Based on ISO standard classification (ISO 22412:2017), the particles of GO/NS and GO/NS/AgNPs were monodispersed.

The accelerated surface area and porosimetry system was applied to measure the BET surface area and the porosity of GO/NS and GO/NS/AgNPs. The data are represented in Table 1. The BET surface area of GO/NS was higher than that of GO/NS/AgNPs. It was possible that the AgNPs blocked the pores of GO/NS and caused a reduction in pore volume and pore size. The data also showed that the BJH adsorption pore volume (0.11 cm^3^/g) and pore size (7.77 nm) of GO/NS were higher than that of GO/NS/AgNPs. Based on the IUPAC classification, both GO/NS and GO/NS/AgNPs are mesoporous materials.

**Figure 9:**
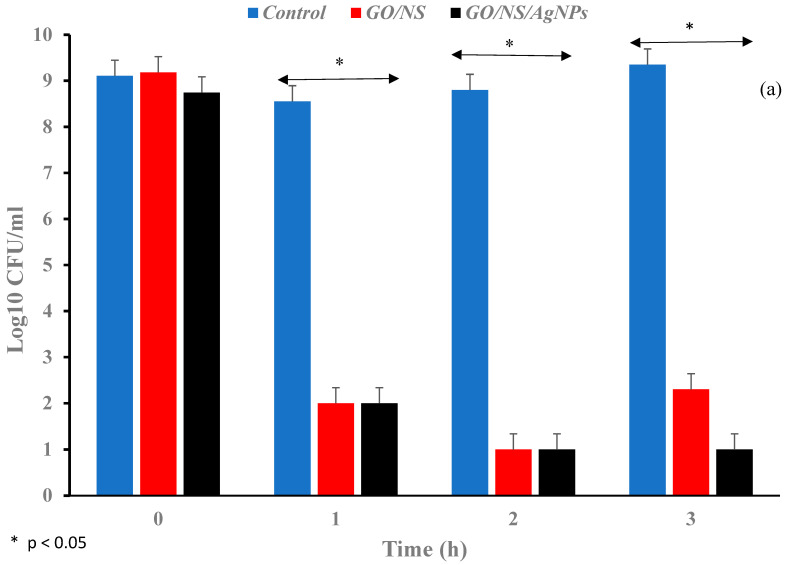

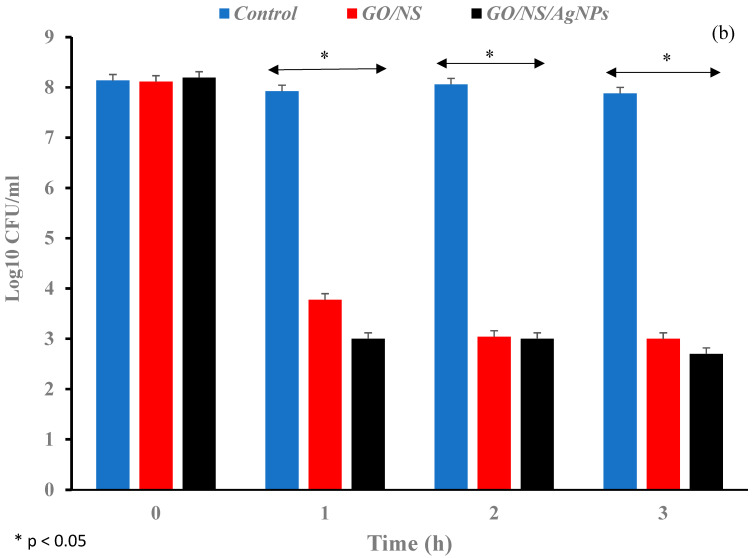
Bar graphs depicting the log reduction against (**a**) *E. coli* and (**b**) *B. subtilis* when exposed to GO/NS and GO/NS/AgNPs. The samples were compared with the control and analyzed at 0 h, 1 h, 2 h, and 3 h.

Figure 9a shows the antibacterial effect over time of GO/NS and GO/NS/AgNPs against *E. coli*. During the first hours, both materials achieved approximately a 7-log reduction of *E. coli*. After two hours, both materials achieved an 8-log reduction. After three hours, GO/NS’s antibacterial effect appeared to reduce and dropped back to 6-log, while GO/NS/AgNPs maintained the 8-log reduction. Based on the ANOVA analysis, both GO/NS and GO/NS/AgNPs were significantly different from the control (F(2,11) = 9.10, *p* = 0.01). These results indicate that GO/NS and GO/NS/AgNPs significantly reduced the concentration of *E. coli*. The average *E. coli* log reduction of GO/NS/AgNPs was higher than that of GO/NS after 3 h.

In Figure 9b, the log reduction of *B. subtilis* for GO/NS (4.3-log) was lower than that of GO/NS/AgNPs (5.2-log) after an hour. After 3 h, the log reduction of GO/NS/AgNPs against *B. subtilis* was higher than that of GO/NS. There was a significant difference in the *B. subtilis* concentration between the composites and control (F(2,11) = 8.71, *p* = 0.02).

When the effectiveness of both materials was compared against *E. coli* and *B. subtilis*, the results indicated that the inactivation of *B. subtilis* was significantly lower than the inactivation of *E. coli* (F(5,23) = 8.34, *p* = 0.0006). Bacteria tend to form spores to help them survive in harsh environmental conditions. It has been shown in previous studies that the spores of *B. subtilis* have a high resistance to radiation, heat, and chemicals [44]. The sturdiness of *B. subtilis* might suggest the difference in log reduction results between *B. subtilis* and *E. coli* when exposed to GO/NS and GO/NS/AgNPs. This will attract further future studies to explain the phenomenon. These results demonstrated the potential of GO/NS and GO/NS/AgNPs in sterilizing resistant bacterial strains.

From Figure 5, the results indicate that more than 99% of *E. coli* and *B. subtilis* populations were killed by GO/NSN and GO/NSN/AgNPs within one hour. Table 2 shows the bacterial log reduction difference between graphene oxide, graphene oxide composites, mesoporous silica, and silver composites. GO/NS and GO/NS/AgNPs both exhibited higher log reduction of Gram-negative and Gram-positive bacteria than other materials within 1 h. A similar result was reported in a study by Nguyen et al. where the material rGO-Ag achieved antibacterial effectiveness against Gram-negative and Gram-positive bacteria after 24 h. Their material took a longer time to achieve effectiveness, possibly due to the loss of functional groups on the surface of the graphene oxide [45]. In the Nguyen et al. study, it was observed that the graphene oxide lost the C=O and O-H carboxyl group after being reduced [45].

Table 3 indicates the inhibition capabilities of GO/NS and GO/NS/AgNPs against *E. coli* and B. *Subtilis*. The inhibition effect of GO/NS/AgNPs was higher than GO/NS. Both composites showed a stronger inhibition effect against *B. Subtilis* than that against *E. coli*. It was demonstrated in the previous study that the silica nanoparticles significantly boosted the antibacterial property of silver nanoparticles [46]. These results highly support the capability of the composites in suppressing the growth of pathogens.

In a Bhargav et al. study, the inhibition zone diameters of antibiotics, including Cefixime, Cefotaxime, Gatifloxacin, and Levofloxacin, against Gram-negative bacteria were 20.76 mm, 25.04 mm, 25.10 mm, and 22.71 mm, respectively [52]. In another study, the amoxicillin exhibited inhibition zones of 35.50 mm and 39.40 mm against some Gram-positive bacteria such as *Streptococcus aureus* and *Bacillus subtilis* [53]. When comparing the results of this literature with Table 3, it showed a promising potential of GO/NS and GO/NS/AgNPs for various future biomedical applications including dental filling, wound treatment, and medical coating [54,55].

## 4. Conclusions

The novel GO/NS/AgNPs composite was successfully synthesized and compared with the GO/NS. The GO/NS/AgNPs contained 3% of silver nanoparticles attached to the nanosilica grown over graphene oxide. The composites in this study showed extraordinarily bacterial inactivation over time. Both GO/NS/AgNPs and GO/NS achieved more than 99% antibacterial efficiency against *E. coli* and *B. subtilis*. Through the zone of inhibition studies, it is highly suggested that GO/NS/AgNPs have a high potential to be applied as an effective antibacterial coating for medical equipment and other surfaces. The composite may become attractive for future biocompatibility studies to explore further applications in the medical field.

## Figures and Tables

**Table 1:** Brunauer–Emmett–Teller (BET) surface areas, Barret–Joyner–Halenda (BJH) pore volumes, and BJH pore sizes of GO/NS and GO/NS/AgNPs.

Composite	BET Surface Area (m^2^/g)	BJH Adsorption Pore Volume (cm^3^/g)	BJH Adsorption Pore Size (nm)
GO/NS	62.09	0.11	7.77
GO/NS/AgNPs	25.83	0.04	7.08

**Table 2:** Bacterial log reduction comparison of different materials from various studies.

Materials	Time Length	Log Reduction (Gram-Negative Bacteria)	Log Reduction (Gram-Positive Bacteria)	Reference
rGO-Ag ^1^	24 h	5	5	[45]
rGO-nAg ^1^	2–2.5 h	1.31	1	[46]
rGO ^2^	2–2.5 h	0.4	0.4	[46]
nAg	2–2.5 h	0.4	0.4	[46]
AgNC-MSNs ^3^	12 h	5.2	3.5	[47]
Ag/PVAGr ^4^	1 h	5	~3	[48]
TA-GA ^5^	4 h	Less than 0.5	4	[49]
GO ^6^	3 h	0.5–1	NA	[50]
GO/CeO_2_^7^	3 h	6	NA	[50]
Silver coated PMMA ^8^ microsphere	24 h	4	4	[51]
GO/NS	1 h	~7	4.3	This work
GO/NS/AgNPs	1 h	~7	5.2	This work

^1^ Reduced graphene oxide–silver nanoparticles, ^2^ reduced graphene oxide, ^3^ silver nanoclusters decorated mesoporous silica nanoparticles, ^4^ silver/polyvinyl alcohol/graphene, ^5^ tannic acid–graphene aerogel, ^6^ graphene oxide, ^7^ graphene oxide–cerium oxide nanoparticles, and ^8^ poly(methylmethacrylate).

**Table 3:** Zone of inhibition diameter (mm) of GO/NS and GO/NS/AgNPs against *E. coli* and *B. subtilis.*

Species	Zone of Inhibiton Diameter (mm)	
	GO/NS	GO/NS/AgNPs
*E. coli*	70 ± 1.0	90 ± 1.0
*B. subtilis*	80 ± 1.0	100 ± 1.0

## Data Availability

The data presented in this study are available on request from the corresponding author.

## Supplementary Materials

The following supporting information can be downloaded at: https://www.mdpi.com/article/10.3390/nano12193341/s1, Figure S1: Examples of Colony Forming Unit (CFU) plate count images of (A) The control *E. coli* samples from 0 h to 3 h, (B) The *E. coli* +GO/NS from 0 h to 3 h, (C) The *E. coli* + GO/NS/AgNPs from 0 h to 3 h; Figure S2: Examples of Colony Forming Unit (CFU) plate count images of (A) The control *B. subtilis* samples from 0 h to 3 h, (B) The *B. Subtilis* + GO/NS from 0 h to 3 h, (C) The *B. subtilis* + GO/NS/AgNPs from 0 h to 3 h; Figure S3: Examples of Kirby-Bauer disk diffusion images of (A) GO/NS and GO/NS/AgNPs against *E. coli*, (B) GO/NS and GO/NS/AgNPs against *B. subtilis*.
