# Supplementary material for: Silver Nanoparticles Functionalized Nanosilica Grown over Graphene Oxide for Enhancing Antibacterial Effect
Source: Nanomaterials (Basel). 2022 Sep 25;12(19):3341. doi: 10.3390/nano12193341 (PMC9565893; doi:10.3390/nano12193341)
Supplement: Supplementary file 1 [file nanomaterials-12-03341-s001.zip › nanomaterials-1898031-supplementary.pdf]

nanomaterials

Supplementary Materials

Silver Nanoparticles Functionalized Nanosilica Grown over Graphene Oxide for Enhancing Antibacterial Effect

Qui Quach and Tarek M. Abdel-Fattah *

Applied Research Center at Thomas Jefferson National Accelerator Facility, Department of Molecular Biology and Chemistry, Christopher Newport University, Newport News, VA 23606, USA
* Correspondence: fattah@cnu.edu

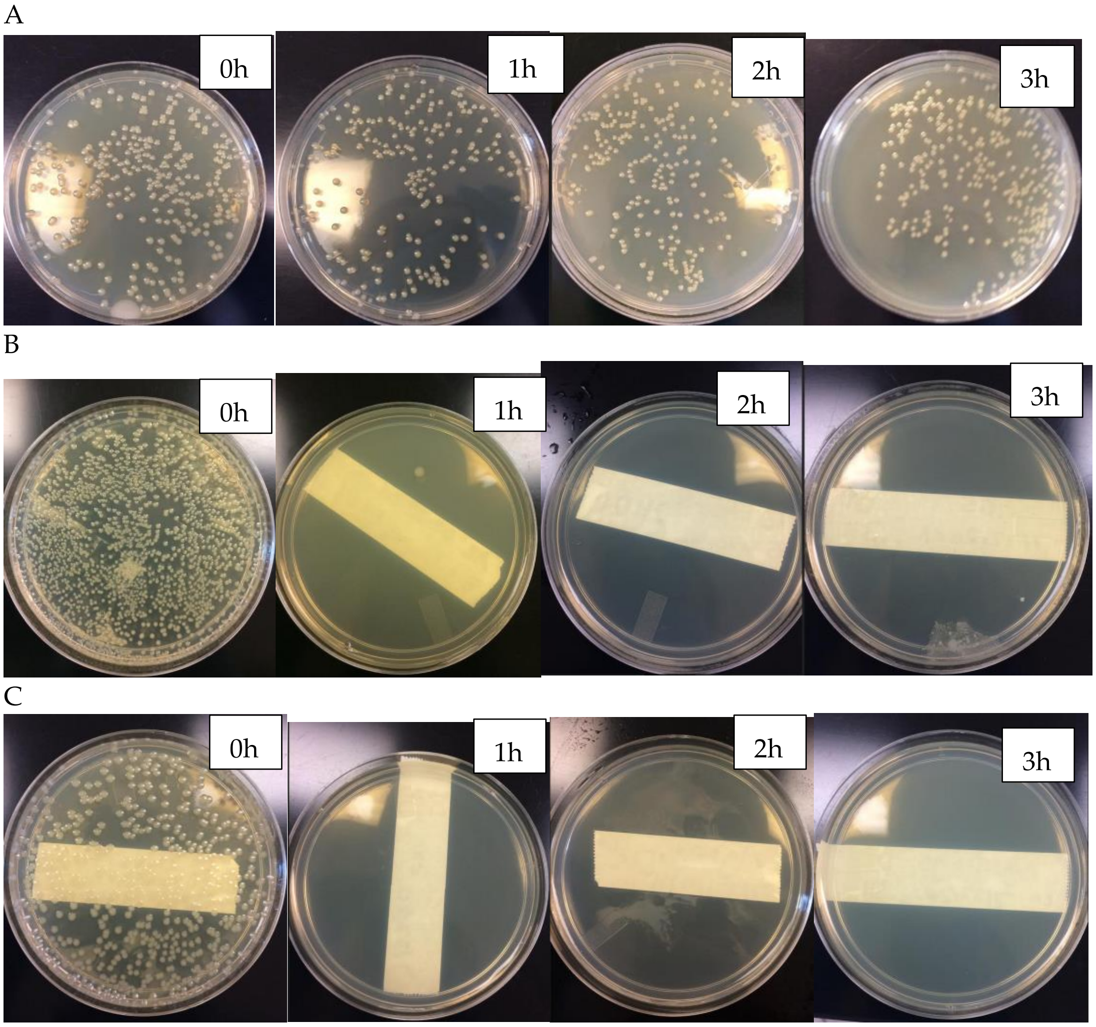

Figure S1: Examples of Colony Forming Unit (CFU) plate count images of A) The control *E.coli* samples from 0h to 3h, B) The *E.coli* +GO/NS from 0h to 3h, C) The *Ecoli*+GO/NS/AgNPs from 0h to 3h.

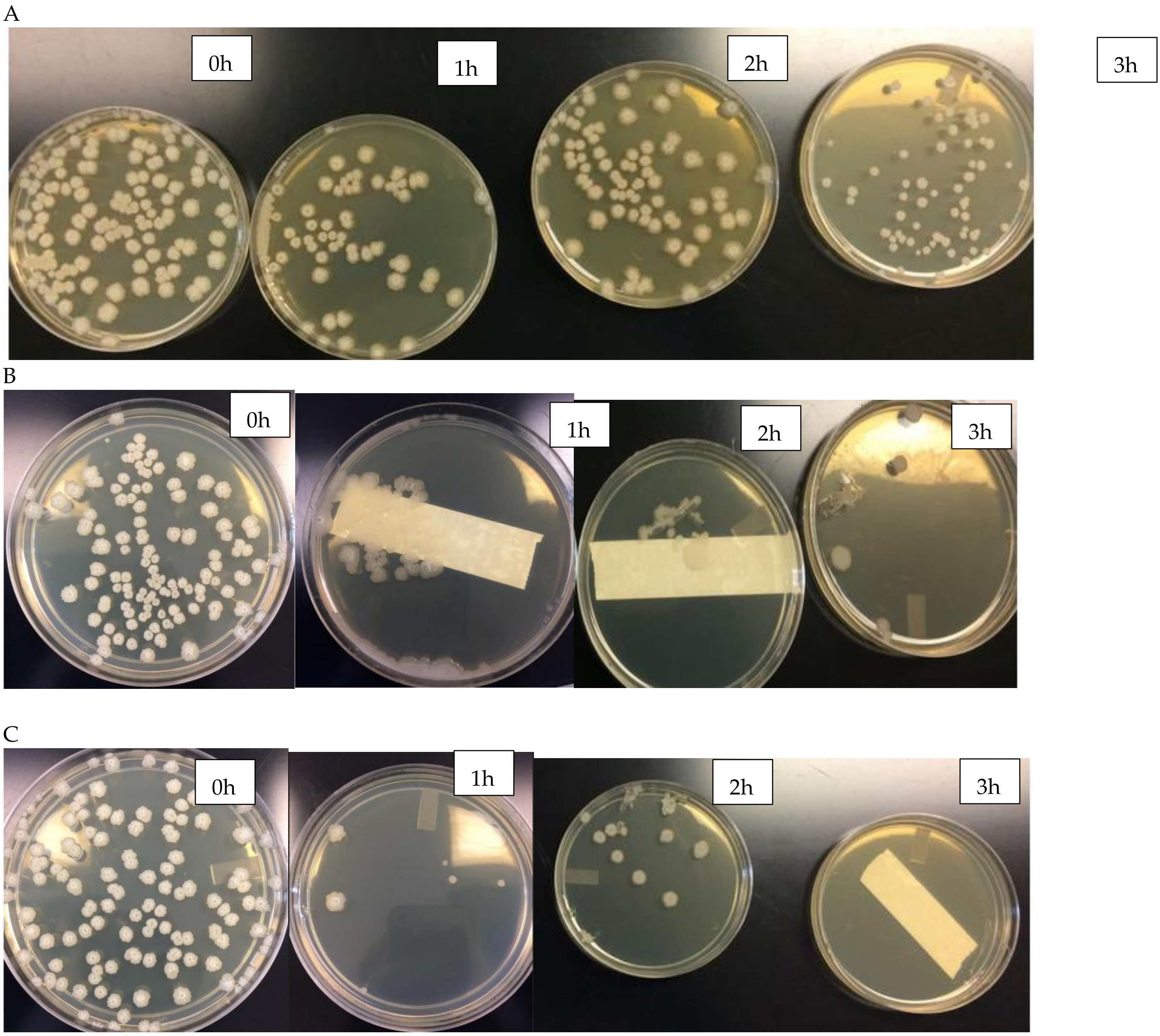

Figure S2: Examples of Colony Forming Unit (CFU) plate count images of A) The control *B.subtilis* samples from 0h to 3h, B) The *B.Subtilis* +GO/NS from 0h to 3h, C) The *B.subtilis* +GO/NS/AgNPs from 0h to 3h.

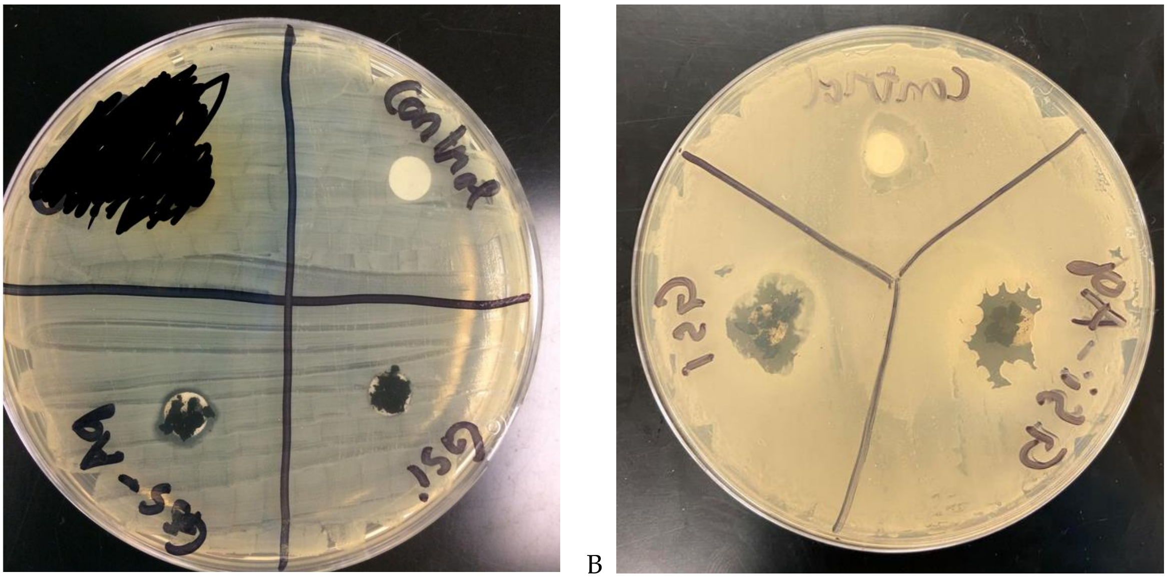

A B

Figure S3: Examples of Kirby-Bauer disk diffusion images of A) GO/NS and GO/NS/AgNPs against *E.coli*, B) GO/NS and GO/NS/AgNPs against *B.subtilis*
